# Risk of mini-mental state examination (MMSE) decline in the elderly with type 2 diabetes: a Chinese community-based cohort study

**DOI:** 10.1186/s12902-020-00606-4

**Published:** 2020-08-25

**Authors:** Lei Zhao, Chao Han, Zheng Zheng, Shuang Ling Xiu, Piu Chan

**Affiliations:** 1 Department of Endocrinology, Beijing Institute of Geriatrics, Xuanwu Hospital of Capital Medical University, Beijing, China; 2 National Clinical Research Center for Geriatric Disorders, Xuanwu Hospital of Capital Medical University, 45 Changchun Road, Xicheng District, Beijing, China; 3 Department of Neurobiology, Geriatrics, and Neurology, Xuanwu Hospital, No. 45 Changchun St., Xicheng District, Beijing, China; 4 Department of Neurology, Beijing Institute of Geriatrics, Xuanwu Hospital of Capital Medical University, Beijing, 100053 China; 5 Parkinson’s Disease Center of Beijing Institute for Brain Disorders, Beijing, 100053 China; 6 Key Laboratory on Neurodegenerative Disease of Ministry of Education, Beijing, 100053 China; 7 Beijing Key Laboratory for Parkinson’s Disease, Beijing, 100053 China; 8 National Clinical Research Center for Geriatric Disorders, Beijing, 100053 China

**Keywords:** Diabetes mellitus, Type 2, Cognitive dysfunction, Aged, Glycated hemoglobin a, Risk factors

## Abstract

**Background:**

We aimed to investigate the correlation of factors involved in the change of Mini-Mental State Examination (MMSE) and type 2 diabetes in the elderly.

**Methods:**

This study was a secondary analysis of a prospective cohort study. Type 2 diabetes patients aged > 55 years were recruited and assigned into three groups based on their glycated hemoglobin (HbA1c) levels: HbA1c < 7, 7% ≤ HbA1c < 8% and HbA1c ≥8%. MMSE decline was considered the endpoint. Factors related to MMSE decline were identified by univariate and multivariate regression analyses.

**Results:**

Altogether, 1519 subjects were included, 883 in the Low group, 333 in the Median group, and 303 in the High group. Age ≥ 75 years, education below elementary school level, not participating in seminars or consultation on healthcare, physical activity less than 30 min/day, cerebrovascular disease history, MMSE score at baseline, and HBA1c ≥8% were associated with cognitive decline by univariate and multivariate analysis. When the other factors were adjusted for, HBA1c ≥8% was independently associated with the severity of cognitive decline (β = 0.58, 95%CI:0.06–1.11, *P* = 0.029) and the occurrence of cognitive decline (odds ratio (OR) = 1.55, 95%CI:1.13–2.12, *P* = 0.007).

**Conclusions:**

In elderly patients with type 2 diabetes, HbA1c ≥8% is an independent factor for cognitive decline and is also associated with the severity of the cognitive decline.

## Background

The number of people with diabetes mellitus is increasing rapidly and has more than doubled globally in thirty years, making it a major public health challenge for all nations [1]. Around 90% of cases of diabetes are type 2 diabetes mellitus (T2DM), and this is particularly important in China, which is an epicenter for the global T2DM epidemic [2]. T2DM is associated with a wide range of complications, including cardiac diseases, diabetic foot, and diabetic nephropathy [3]. However, the adverse impact of T2DM is not limited to these common diseases, and it is also associated with exacerbation of age-related decline in physical and cognitive function [4].

Individuals with T2DM score less well on cognitive function tests such as the Mini-Mental State Examination (MMSE) than those without diabetes [5], and a clear relationship between diabetes in midlife and greater cognitive decline has been demonstrated [6, 7]. Although the association in elderly patients is less clear because of the small numbers investigated [8].

Hemoglobin A1C (HbA1c) or glycated hemoglobin represents a measure of the blood glucose levels over the previous three months. HbA1c can evaluate glucose control in individuals with diagnosed diabetes and can also be used in diagnosing diabetes and people at risk of developing diabetes [9]. HbA1c is associated with cognitive decline [10]. Most, but not all [11], cross-sectional studies associate higher levels of HbA1c with worse cognitive function in individuals with T2DM [12, 13]. This suggests that poor glycemic control is an important factor related to cognitive decline. But the studies using high HbA1c levels to measure this have shown some conflicting results [4, 14–17]. Therefore, at the moment, the association between HbA1c and cognitive decline in patients with T2DM remains unclear. A study in elderly patients found diabetes, poor glycemic control, and longer diabetes duration were all associated with incident cognitive impairment. However, in persons with well-controlled diabetes, as assessed by HbA1c, there was not a significantly higher risk of cognitive impairment compared with persons without diabetes [17]. Another study in middle-aged and young older adults with T2DM suggested that HbA1c over 7 years of follow-up was not associated with cognitive function [4]. These contradictions between studies suggest that other factors may also influence whether glycemic control is associated with cognitive impairment.

If HbA1c is an important indicator of cognitive decline, this suggests that improving glycemic control in elderly patients is vital to decrease the risk of cognitive decline. However, understanding other factors that are also involved would help target the patients most at risk of cognitive decline. Therefore, the aim of this study was to investigate the association of HbA1c levels with MMSE decline in elderly patients with T2DM and find the correlative factors involved.

## Methods

### Study design and patients

This was a retrospective analysis of a community-based cohort study (BLSA II) [18], which included the patients with T2DM from 3 urban districts (Xicheng, Dongcheng, and Xuanwu) and 1 outer suburban area (Shunyi) of Beijing. The baseline data were collected from August 2010 to January 2011, and the last follow-up was undertaken in April 2014. The inclusion criteria were as follows: 1) aged > 55 years; 2) diagnosed with T2DM that was under diet control or medication [9]. The exclusion criteria were: 1) acute critical illness or severe psychiatric diseases that would affect the scale evaluation such as dementia and depression; 2) blind, deaf, dumb, and vegetative; 3) complicated with severe diseases including heart, liver, renal diseases and malignant tumors. The patients were assigned into three groups according to their baseline HbA1c levels: HbA1c < 7, 7% ≤ HbA1c < 8% and HbA1c ≥8%.

The study was approved by the research ethics committee of Xuanwu Hospital of Capital Medical University before study initiation. Written informed consent was obtained from all participants for the original data collection.

### Clinical data collection and examination method

The baseline information of the subjects was collected, including sex, age, smoking, alcohol consumption, education, whether they lived alone, income, physical exercise, dietary habits, intake of fruit and vegetables, body mass index (BMI), history of cerebrovascular disease (CED), history of cardiovascular disease (CVD) and peripheral vascular disease, history of hypertension, participation in community-based healthcare seminars and consultations, alanine aminotransferase (ALT), triglyceride (TG), total cholesterol (TC), high-density lipoprotein (HDL), low-density lipoprotein (LDL), glucose (GLU), urinary albumin (UA), creatinine (Cr), apolipoprotein E (ApoE), and HbA1c. The biochemical analysis was performed by a Hitachi 7600 automatic biochemical analyzer (Hitachi high-tech, Fukuoka, Japan). The HbA1c level was determined by a Bio-Rad VARIANT™ II TURBO Hemoglobin Testing System for HbA1c when baseline information was collected.

#### Assessment of cognitive function

The patient’s cognitive function was evaluated by the Chinese edition of the Mini-Mental State Examination (MMSE) [19]. The MMSE was invented in 1975 and since then has been widely used as a scale for dementia. The total score was 30 and assessed by the raw MMSE values stratified threshold based on education years, which was validated by previous research. MMSE was examined at baseline as well as 3 years later during the follow-up.

### Definitions and follow up

The change of MMSE was defined as the difference between baseline and follow-up values after 3 years.

The last follow up occurred between November 2013 and April 2014 in the form of a questionnaire in the community hospitals.

### Statistical analysis

The data analysis was performed using R software (version 3.5.1; R Development Core Team 2018, www.R-project.org). The continuous variables that followed normal distribution were represented as the mean ± standard deviation (SD). The continuous variables that did not follow normal distribution were represented by median (range or interquartile range (IQR)). The categorical variables were represented as n (%). The continuous variables that follow normal distribution were examined by t-test or analysis of variance (ANOVA). The continuous variables that did not follow normal distribution were tested by the Mann-Whitney U or Kruskal-Wallis test. The categorical variables were tested by the χ^2^ test or Fisher exact test. The association analysis was performed by univariate or multivariate regression. A *P* < 0.05 value was considered statistically significant.

## Results

### Baseline characteristics

The study included 1519 subjects. Their mean age was 72 ± 6.3 years old. Among them, 580 (38.2%) were male, and 939 (61.8%) were female. The numbers of subjects in the three groups were 883 in the Low group, 333 in the Median group, and 303 in the High group. The baseline information collected from the patients is shown in Table 1. Age, gender, and most of the clinical indexes were similar between the groups except GLU (*P* < 0.001), HbA1c (P < 0.001), TGP = 0.01), and UA (*P* = 0.005). The number of patients educated below elementary school level (*P* = 0.003), BMI (*P* = 0.001), and MMSE variation (*P* = 0.022) but not the MMSE scores at baseline or follow-up were also different between the groups.

**Table 1:** Baseline information of the patients with type 2 diabetes mellitus included in the study

Characteristics	Total (*n* = 1519)	Low group HbA1c < 7% (*n* = 883)	Median group 7% ≤ HbA1c < 8% (*n* = 333)	High group HbA1c ≥8% (*n* = 303)	P
Age (mean ± SD), years	72 ± 6.3	71.8 ± 6.3	72.3 ± 6.3	72.1 ± 6	0.371
Age, years, n (%)					
55–64	249 (16.4)	155 (17.6)	52 (15.6)	42 (13.9)	0.483
65–74	723 (47.6)	413 (46.8)	155 (46.5)	155 (51.2)	
≥ 75	547 (36)	315 (35.7)	126 (37.8)	106 (35)	
Cr	70.2 ± 26.2	70.3 ± 30.1	70.9 ± 19	69.2 ± 20.4	0.701
GLU	6.7 ± 2.1	5.8 ± 0.9	7 ± 1.1	9.2 ± 3.2	< 0.001
HbA1c	7 ± 1.6	6 ± 0.6	7.4 ± 0.3	9.5 ± 1.6	< 0.001
HDL	1.3 ± 0.3	1.3 ± 0.3	1.3 ± 0.3	1.3 ± 0.3	0.361
LDL	2.9 ± 0.7	2.9 ± 0.8	2.9 ± 0.7	2.9 ± 0.8	0.971
TC	5.2 ± 1	5.2 ± 1.1	5.2 ± 1	5.2 ± 1	0.965
TG	1.7 ± 1	1.6 ± 0.9	1.7 ± 0.8	1.8 ± 1.4	0.01
UA	327.1 ± 89.7	326.3 ± 86.5	339.3 ± 95	316.1 ± 91.5	0.005
ApoE23	1078 (90.4)	621 (91.2)	241 (90.9)	216 (87.4)	0.218
ApoE4	115 (9.6)	60 (8.8)	24 (9.1)	31 (12.6)	
Sex					
male	580 (38.2)	345 (39.1)	126 (37.8)	109 (36)	0.625
female	939 (61.8)	538 (60.9)	207 (62.2)	194 (64)	
Education					
Elementary school and below	534 (36.1)	280 (32.6)	137 (42.2)	117 (39.9)	0.003
High school and above	944 (63.9)	580 (67.4)	188 (57.8)	176 (60.1)	
Live Alone					
yes	104 (6.9)	64 (7.3)	23 (6.9)	17 (5.6)	0.619
no	1413 (93.1)	818 (92.7)	309 (93.1)	286 (94.4)	
income					
The monthly per capita income in the family ≤3000	1098 (79.6)	643 (80.8)	235 (77.3)	220 (78.9)	0.413
The monthly per capita income in the family > 3000	281 (20.4)	153 (19.2)	69 (22.7)	59 (21.1)	
Participation in healthcare seminar/consulting at least once a month					
yes	902 (61.2)	534 (62.4)	195 (59.8)	173 (59.2)	0.539
no	572 (38.8)	322 (37.6)	131 (40.2)	119 (40.8)	
smoke					
no	1361 (89.6)	795 (90)	303 (91)	263 (86.8)	0.181
yes	158 (10.4)	88 (10)	30 (9)	40 (13.2)	
drink					
no	1334 (87.8)	774 (87.7)	287 (86.2)	273 (90.1)	0.313
yes	185 (12.2)	109 (12.3)	46 (13.8)	30 (9.9)	
Physical Activity					
Exercise less than 30 min/day	363 (24.6)	218 (25.3)	78 (24)	67 (22.9)	0.671
Exercise more than 30 min/day	1115 (75.4)	642 (74.7)	247 (76)	226 (77.1)	
High-protein diet					
High-protein diet<=1 item	276 (18.2)	160 (18.1)	50 (15)	66 (21.8)	0.087
High-protein diet: 2–3 items	1243 (81.8)	723 (81.9)	283 (85)	237 (78.2)	
Intake of fruits/vegetables more than twice a day					
no	165 (10.9)	90 (10.2)	33 (9.9)	42 (13.9)	0.171
yes	1354 (89.1)	793 (89.8)	300 (90.1)	261 (86.1)	
BMI					
normal	534 (35.2)	346 (39.2)	98 (29.4)	90 (29.7)	0.001
Overweight or obesity	984 (64.8)	536 (60.8)	235 (70.6)	213 (70.3)	
CVD History					
no	748 (49.2)	417 (47.2)	166 (49.8)	165 (54.5)	0.092
yes	771 (50.8)	466 (52.8)	167 (50.2)	138 (45.5)	
CED History					
no	1131 (74.5)	646 (73.2)	244 (73.3)	241 (79.5)	0.077
yes	388 (25.5)	237 (26.8)	89 (26.7)	62 (20.5)	
HP History					
no	128 (8.4)	85 (9.6)	26 (7.8)	17 (5.6)	0.085
yes	1391 (91.6)	798 (90.4)	307 (92.2)	286 (94.4)	
MMSE score (baseline)	28.5 ± 2.2	28.5 ± 2.2	28.4 ± 2.2	28.6 ± 2.4	0.51
MMSE score (follow-up)	27.3 ± 3.6	27.4 ± 3.3	27.3 ± 3.9	26.9 ± 3.8	0.086
MMSE variation	−1.2 ± 3.5	−1.1 ± 3.3	−1.1 ± 3.6	−1.7 ± 3.6	0.022

Abbreviations: triglyceride (TG), total cholesterol (TC), high-density lipoprotein (HDL), low-density lipoprotein (LDL), glucose (GLU), urinary albumin (UA), creatinine (Cr), apolipoprotein E (ApoE), glycated hemoglobin (HbA1c), body mass index (BMI), cardiovascular disease (CVD), cerebrovascular disease (CED), hypertension (HP), mini-mental state examination (MMSE)

### Linear regression analysis of factors related to the severity of the decline in MMSE score

Univariate analysis of factors related to a decline in MMSE score (Table 2), identified age ≥ 75 years (Beta = 0.97 95% confidence interval (CI):0.42–1.51, *P* = 0.001), education below elementary school level (Beta = 1.17 95%CI:0.75–1.59, *P* < 0.001), not participating in seminars or consultation on healthcare (Beta = 0.43 95%CI:0.08–0.78, *P* = 0.017), physical activity more than 30 min/day (Beta = − 0.4 95%CI:-0.8–0, *P* = 0.05), CED history (Beta = 0.49 95%CI:0.1–0.89, *P* = 0.015), MMSE score at baseline (Beta = 0.56 95%CI:0.47–0.64, *P* < 0.001), and HBA1c ≥8% (Beta = 0.61 95%CI:0.08–1.14, *P* = 0.024).

**Table 2:** Univariate regression analysis for factors related to MMSE decline in elderly patients with type 2 diabetes mellitus

Characteristics	Beta (95%CI)	*P*-value
Female	0.15 (− 0.29–0.59)	0.505
Age, years		
55–64	1	
65–74	− 0.06 (− 0.56–0.43)	0.798
≥ 75	0.97 (0.42–1.51)	0.001
Elementary school and below	1.17 (0.75–1.59)	< 0.001
Lives alone, no	0.44 (−0.25–1.13)	0.208
Participation in healthcare seminars/consultation at least once a month, no	0.43 (0.08–0.78)	0.017
Smoker	0.03 (−0.59–0.65)	0.919
Drinking alcohol	−0.18 (− 0.76–0.41)	0.55
Physical Activity of more than 30 min/day	−0.4 (− 0.8–0)	0.05
High-protein diet: 2–3 items	0.3 (− 0.15–0.75)	0.197
Intake of fruits/vegetables more than twice a day, yes	0.08 (− 0.48–0.64)	0.773
Overweight or obese	−0.11 (− 0.48–0.26)	0.555
CVD History, yes	0.05 (− 0.3–0.39)	0.793
CED History, yes	0.49 (0.1–0.89)	0.015
HP History, yes	−0.23 (− 0.86–0.4)	0.476
MMSE score (baseline)	0.56 (0.47–0.64)	< 0.001
TG	−0.04 (− 0.26–0.17)	0.7
TC	0.08 (−0.41–0.57)	0.75
HDL	−0.21 (− 0.92–0.51)	0.571
LDL	−0.19 (− 0.81–0.44)	0.56
UA	0 (0–0)	0.972
Cr	0.01 (0–0.01)	0.099
HbA1c		
7% ≤ HbA1c < 8%	1	
HbA1c < 7%	0.05 (−0.37–0.48)	0.808
HbA1c ≥8%	0.61 (0.08–1.14)	0.024

*Abbreviations*: *CI* confidence interval, *TG* triglyceride, *TC* total cholesterol, *HDL* high-density lipoprotein, *LDL* low-density lipoprotein, *UA* urinary albumin, *Cr* creatinine, *HbA1c* glycated hemoglobin, *CVD* cardiovascular disease, *CED* cerebrovascular disease, *HP* hypertension, *MMSE* mini-mental state examination

Factors that had a level at or close to significance (*P* < 0.1) were then included in multivariate analysis for independent factors related to MMSE decline (Table 3). This showed that age ≥ 75 years (Beta = 0.99 95%CI:0.45–1.52, *P* < 0.001), education to elementary school and below (Beta = 1.18 95%CI:0.78–1.58, *P* < 0.001, not participating in healthcare seminars or consultation at least once a month (Beta = 0.42 95%CI:0.07–0.77, *P* = 0.018), physical activity for more than 30 min/day (Beta = − 0.44 95%CI:-0.83--0.05, *P* = 0.029), a CED history (Beta = 0.48 95%CI:0.09–0.87, *P* = 0.015), MMSE score at baseline (Beta = 0.57 95%CI:0.49–0.65, *P* < 0.001), and HbA1c ≥8% (Beta = 0.58 95%CI:0.06–1.11, P = 0.029) all remained significant factors.

**Table 3:** Multivariate analysis of factors independently associated with MMSE decline value in elderly patients with type 2 diabetes mellitus

Characteristics	Beta 95%CI	P-value
Age, years		
55–64	1	
65–74	−0.05 (−0.54–0.44)	0.841
≥ 75	0.99 (0.45–1.52)	< 0.001
Elementary school and below	1.18 (0.78–1.58)	< 0.001
Participation in healthcare seminar/consulting at least once a month, no	0.42 (0.07–0.77)	0.018
Physical activity for more than 30 min/day	−0.44 (−0.83--0.05)	0.029
CED History, yes	0.48 (0.09–0.87)	0.015
MMSE score (baseline)	0.57 (0.49–0.65)	< 0.001
HbA1c		
7% ≤ HbA1c < 8%	1	
HbA1c < 7%	0.05 (−0.37–0.47)	0.807
HbA1c ≥8%	0.58 (0.06–1.11)	0.029

*Abbreviations*: *CED* cerebrovascular disease, *MMSE* mini-mental state examination, *HbA1c* glycated hemoglobin

### Logistic regression analysis of factors related to the decline in MMSE score

When logistic regression was used for analysis of factors related to a decline in MMSE score (Table 4), the univariate analysis identified age ≥ 75 years (odds ratio (OR) = 1.66 95% CI:1.26–2.19, *P* < 0.001), physical activity than 30 min/day (OR = 0.77 95%CI:0.62–0.92, *P* = 0.022), MMSE score at baseline (OR = 1.41 95%CI:1.35–1.49, *P* < 0.001), LDL (OR = 0.85 95%CI:0.75–0.96, *P* = 0.01) and HBA1c ≥8% (OR = 1.52 95%CI:1.13–2.03, *P* = 0.005).

**Table 4:** Univariate logistic regression analysis of MMSE decline

Characteristics	OR_95CI	P_value
Female	0.94 (0.77–1.14)	0.508
Age, years		
55–64	1	
65–74	1.13 (0.87–1.48)	0.363
≥ 75	1.66 (1.26–2.19)	< 0.001
Elementary school and below	0.83 (0.68–1.01)	0.069
Lives alone, no	1.3 (0.9–1.88)	0.162
Participation in healthcare seminars/consultation at least once a month, no	1.09 (0.89–1.32)	0.402
Smoker	1.07 (0.79–1.46)	0.648
Drinking alcohol	0.97 (0.73–1.29)	0.837
Physical Activity of more than 30 min/day	0.77 (0.62–0.96)	0.022
High-protein diet: 2–3 items	1.25 (0.98–1.6)	0.066
Intake of fruits/vegetables more than twice a day, yes	0.75 (0.55–1.01)	0.061
Overweight or obese	0.93 (0.76–1.13)	0.443
CVD History, yes	1.06 (0.88–1.27)	0.57
CED History, yes	1.16 (0.94–1.44)	0.177
HP History, yes	1.16 (0.83–1.63)	0.373
MMSE score (baseline)	1.41 (1.35–1.49)	< 0.001
TG	0.99 (0.9–1.08)	0.773
TC	0.92 (0.84–1.01)	0.067
HDL	0.82 (0.61–1.12)	0.21
LDL	0.85 (0.75–0.96)	0.01
UA	1 (1–1)	0.629
Cr	1 (1–1.01)	0.063
HbA1c		
7% ≤ HbA1c < 8%	1	
HbA1c < 7%	1.13 (0.89–1.42)	0.314
HbA1c ≥8%	1.52 (1.13–2.03)	0.005

*Abbreviations*: *CI* confidence interval, *TG* triglyceride, *TC* total cholesterol, *HDL* high-density lipoprotein, *LDL* low-density lipoprotein, *UA* urinary albumin, *Cr* creatinine, *HbA1c* glycated hemoglobin, *CVD* cardiovascular disease, *CED* cerebrovascular disease, *HP* hypertension *MMSE* mini-mental state examination (MMSE)

The multivariate analysis is shown in Table 5. This showed that age ≥ 75 years (OR = 1.97 95%CI:1.43–2.72, *P* < 0.001), education to elementary school and below (OR = 1.91 95%CI:1.48–2.45, P < 0.001, physical activity less than 30 min/day (OR = 0.79 95%CI:0.62–1, *P* = 0.048), a CED history (OR = 1.3 95%CI:1.03–1.64, *P* = 0.029), MMSE score at baseline (OR = 1.56 95%CI:1.47–1.66, P < 0.001), and HbA1c ≥8% (OR = 1.52 95%CI:1.13–2.12, *P* = 0.007) were all significant factors independently associated with decline in MMSE score.

**Table 5:** Multivariate logistic regression analysis of MMSE decline

Characteristics	OR_95CI	P-value
Female	1.19 (0.92–1.55)	0.185
Age, years		
55–64	1	
65–74	1.12 (0.84–1.49)	0.456
≥ 75	1.97 (1.43–2.72)	< 0.001
Elementary school and below	1.91 (1.48–2.45)	< 0.001
Lives Alone, no	1.36 (0.9–2.06)	0.144
Participation in healthcare seminar/consulting at least once a month, no	1.28 (1.04–1.58)	0.022
smoke	1.11 (0.77–1.6)	0.588
drink	0.86 (0.61–1.21)	0.383
PhyAct more than 30 min/day	0.79 (0.62–1)	0.048
High-protein diet: 2–3 items	1.14 (0.87–1.49)	0.344
Intake of fruits/vegetables more than twice a day, yes	0.84 (0.6–1.17)	0.302
Overweight or obesity	0.99 (0.8–1.23)	0.937
CVD His, yes	1.06 (0.86–1.3)	0.601
CED His, yes	1.3 (1.03–1.64)	0.029
HP His, yes	0.92 (0.63–1.34)	0.67
MMSE score (baseline)	1.56 (1.47–1.66)	< 0.001
TG	0.97 (0.85–1.1)	0.644
TC	1.09 (0.81–1.46)	0.585
HDL	0.9 (0.59–1.39)	0.644
LDL	0.84 (0.58–1.23)	0.373
UA	1 (1–1)	0.51
Cr	1 (1–1.01)	0.49
HbA1c		
7% ≤ HbA1c < 8%	1	
HbA1c < 7%	1.18 (0.91–1.51)	0.205
HbA1c ≥8%	1.55 (1.13–2.12)	0.007

Note analysis was by stepwise regression

*Abbreviations*: *CI* confidence interval, *TG* triglyceride, *TC* total cholesterol, *HDL* high-density lipoprotein, *LDL* low-density lipoprotein, *UA* urinary albumin, *Cr* creatinine, *HbA1c* glycated hemoglobin, *CVD* cardiovascular disease, *CED* cerebrovascular disease, *HP* hypertension, *MMSE* mini-mental state examination

### Association between MMSE decline and HbA1c level

**Fig. 1:**
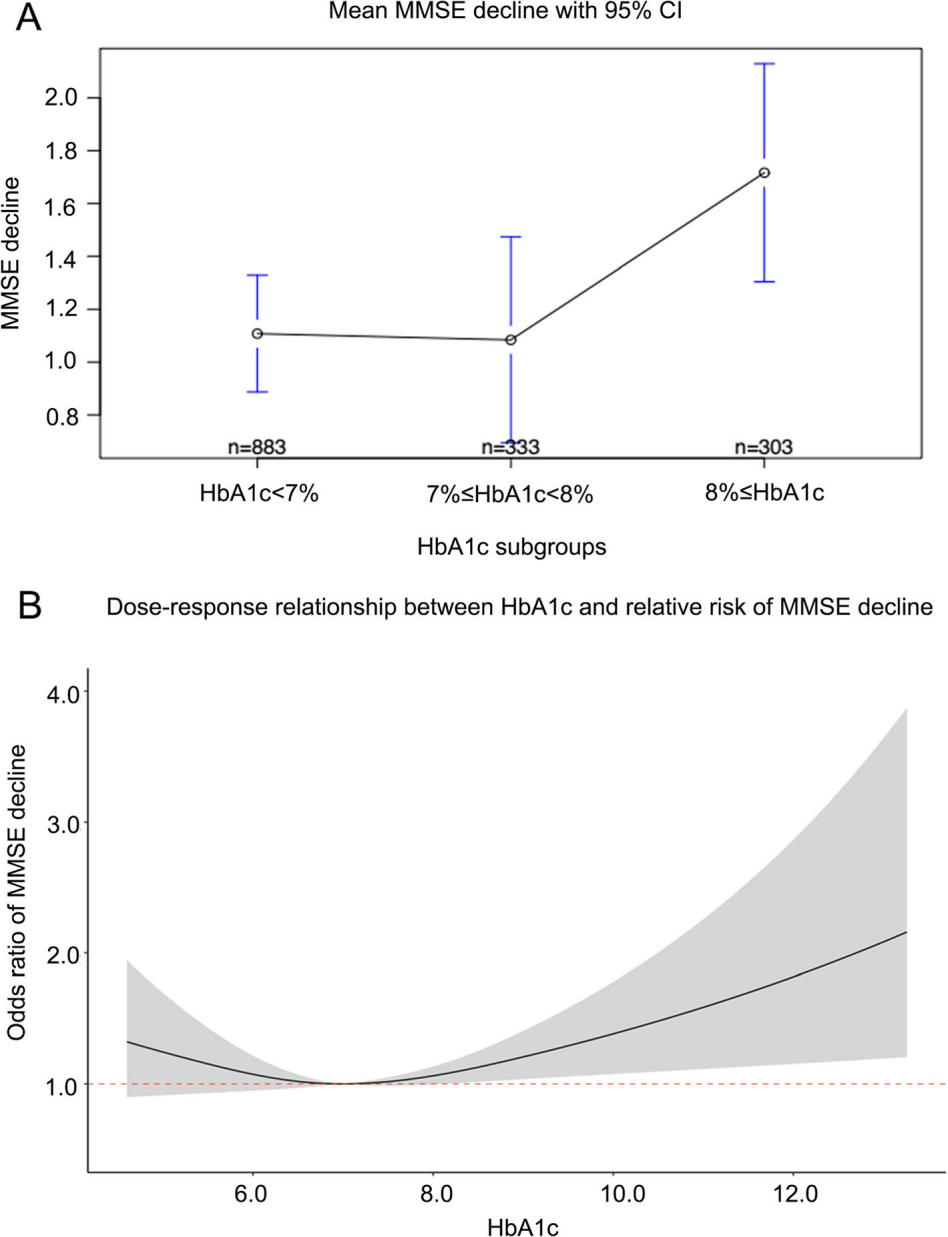
Graphs showing the association of cognitive decline with glycated hemoglobin (HbA1c) levels. **a**: Mini mental state examination (MMSE) decline in the three groups based on HbA1c levels. **b**: Dose response relationship between HbA1c and risk of MMSE decline

Figure 1 shows the association between MMSE decline and HbA1c level. It can be seen that patients with HbA1c levels ≥8% (the High group) showed more decline than those in the other two groups, and the risk of MMSE decline increased above 8%.

## Discussion

The aim of this study was to investigate the association between HbA1c levels and MMSE decline in elderly patients with T2DM. The results show that age ≥ 75 years, education below elementary school level, not participating in seminars or consultation on healthcare, physical activity less than 30 min/day, cerebrovascular disease history, MMSE score at baseline, and HBA1c ≥8% were all associated with MMSE decline by univariate and multivariate analysis. After adjustment for the other factors, HbA1c ≥8% was independently associated with MMSE decline and its severity.

There has been some controversy over whether HbA1c levels as an indicator of glycemic control in patients with T2DM can be used to predict cognitive decline. There is strong evidence that HbA1c is associated with cognitive decline [10]. Most studies suggest higher levels of HbA1c correlate with worse cognitive function in individuals with T2DM [12, 13], but some do not show this correlation [11]. Studies using high HbA1c levels to measure cognitive decline have also shown conflicting results [4, 14–17]. This conflict is likely to arise from other contributing factors. The results of this study showed that a number of factors were related to cognitive decline in elderly patients with T2DM including age ≥ 75 years, education below elementary school level, not participating in seminars or consultation on healthcare, undertaking physical activity for less than 30 min/day, cerebrovascular disease history, and MMSE score at baseline as well as HbA1c ≥8%. However, when the model was adjusted for these factors, this study found that HbA1c ≥8% was independently associated with cognitive decline. This result suggests that active measures should be taken to ensure that elderly patients make every effort to control their glucose levels below 8% to prevent cognitive decline [20, 21]. There are several potential mechanisms for higher glucose levels, increasing the risk of cognitive decline. These include acute hyperglycemia, where very high blood glucose concentrations may cause alterations in cerebral blood flow or osmotic changes in neurons; chronic hyperglycemia, which may cause structural changes in the brain through mechanisms such as cerebral microvascular disease [22, 23]; and insulin resistance, where the failure of brain cells to respond to insulin might result in synaptic, metabolic and immune response impairments. However, while the results of this study concentrated on HbA1c levels, which indicate glucose levels over the previous 3 months, there is evidence indicating that fluctuations or peaks in glucose levels may also be linked to cognitive decline and dementia risk [20, 21]. Glycemic fluctuations may have a greater adverse effect on endothelial function and induce more oxidative stress than sustained hyperglycemia, potentially leading to greater cognitive decline [24]. So, the relationship between glucose control and cognitive decline is likely to be complex, and efforts to prevent glucose fluctuations might also be important. Further study is needed to fully understand the mechanisms involved.

Age is clearly related to the degree of decline in MMSE scores both in populations with and without T2DM [25, 26]. So, the result that age ≥ 75 years was related to a decline in MMSE was consistent with previous studies. Other factors related to MMSE decline in this study may also influence the glycemic control of the patients. For example, education below elementary school level and monthly participation in seminars or consultation on healthcare are both likely to influence whether the patient adheres to their medication or diet to achieve glycemic control. This is supported by another study that found that higher education level was a protective factor for cognitive impairment in elderly patients with T2DM [27]. Physical activity has also been shown by other studies to be an important protective factor against cognitive decline, as summarized in a systematic review [28]. Cardiovascular disease history was also identified as an important factor in this study. This is supported by the established view that cardiovascular risk factors are related to late-life cognitive decline [29]. Both physical activity and cardiovascular risk factors are considered so important that the World Dementia Council (WDC) concluded that regular physical activity and management of cardiovascular risk factors, including diabetes, obesity, smoking, and hypertension, would reduce the risk of cognitive decline [30].

This study has some limitations. The baseline measurement of HbA1c was undertaken at one time point and may not have been a true reflection of the typical value. As a retrospective study, no cause and effect can be deduced from the results. Cognitive function was based on MMSE alone, and other measures of cognitive function may not support the results. We did not have the full data regarding the medication use for all patients, some drugs such as benzodiazepines are known to impair cognitive function, so there may have been some differences in their use between groups that may have influenced the results of the study. The 3-year follow-up might be too short to study a decrease in cognitive functions. But the follow up has continued, and long-term changes of cognitive functions will be analyzed in the future.

## Conclusions

In elderly patients with type 2 diabetes, a number of factors were related to MMSE decline. Nevertheless, the analysis suggested that HbA1c ≥8% was an independent factor for cognitive decline and was also associated with the severity of the cognitive decline.

## Data Availability

Data sharing is not applicable to this article, as no datasets were generated or analyzed during the current study.

## Abbreviations

HbA1c: Glycated hemoglobin
MMSE: Mini-Mental State Examination
T2DM: Type 2 diabetes mellitus
BMI: Body mass index
CED: Cerebrovascular disease
CVD: Cardiovascular disease
ALT: Alanine aminotransferase
TG: Triglyceride
TC: Total cholesterol
HDL: High-density lipoprotein
LDL: Low-density lipoprotein
GLU: Glucose
UA: Urinary albumin
Cr: Creatinine
SD: Standard deviation
IQR: Interquartile range
ANOVA: Analysis of variance
